# Uncertainty-Calibrated Collaborative Filtering via Heteroscedastic Bayesian Neural Networks

**DOI:** 10.3390/e28091025

**Published:** 2026-09-14

**Authors:** Xiaowei Wang

**Affiliations:** The College of Cybersecurity, Shandong University of Political Science and Law, Jinan 250013, China; wangxiaowei@sdupsl.edu.cn

**Keywords:** Bayesian neural networks, collaborative filtering, uncertainty quantification, calibration, cold-start recommendation

## Abstract

Although recommendation systems based on neural collaborative filtering (NCF) can achieve high rating accuracy, the point estimates they generate do not express prediction confidence. Existing variants that account for uncertainty, such as Monte Carlo dropout, deep ensemble models, and single-head Bayesian networks, can only capture epistemic uncertainty. We propose BayesNCF-v2, a single-model Bayesian neural collaborative filtering framework that combines a Bayes-by-Backprop output layer with a heteroscedastic head trained under the Gaussian NLL, thereby jointly learning aleatoric and epistemic uncertainty. Across five seeds and three datasets, compared to standard NCF, it reduces ECE by 25–73% and NLL by 7–20%, while its calibration performance shows no statistically significant difference from heteroscedastic MC dropout and approaches that of a heteroscedastic deep ensemble consisting of five models, despite having significantly fewer parameters. For cold-start users, the learned aleatoric variance increases, thereby reducing the ECE for cold-start users on three datasets. These results hold consistently across both random and temporal partitions, demonstrating that heteroscedastic aleatoric modeling is the main driver of calibration in these experiments.

## 1. Introduction

Neural collaborative filtering (NCF) has become a mainstream paradigm in recommendation systems, achieving powerful rating prediction capabilities by learning user and item embeddings [1]. However, the point estimates generated by standard NCF models do not express prediction confidence. When the model predicts a rating of 4.2 for a cold-start user with only two historical interactions, it assigns the same level of confidence to that prediction as it would to a user with thousands of rating records. This overconfidence poses a problem: downstream systems relying on these predictions lack a signal indicating when they should trust the model.

Bayesian methods provide a principled framework for uncertainty [2,3]. Blundell et al. proposed Bayes by Backprop, a variational training scheme for Bayesian neural networks [3]. Gal and Ghahramani demonstrated that Monte Carlo (MC) dropout can approximate Bayesian inference in deep neural networks [4]. Lakshminarayanan et al. proposed deep ensemble models as a practical alternative for estimating uncertainty through prediction diversity [5]. These ideas have been introduced into the field of collaborative filtering: Bayesian deep collaborative filtering model  [6], ensemble-based Bayesian recommendation systems [7], and probabilistic embeddings [8,9] are all capable of expressing uncertainty.

However, they all share a common limitation: they can only capture model uncertainty caused by limited data, while ignoring the noise inherent in the observations themselves—that is, aleatoric uncertainty. In recommendation systems, aleatoric uncertainty arises naturally because some users’ ratings are relatively stable, while others inherently exhibit rating noise. Some items receive polarized ratings, while others are universally well received.

This distinction is crucial because these two types of uncertainty manifest differently. Kendall and Gal demonstrated in the field of computer vision that obtaining calibrated predictions requires explicit heteroscedastic aleatoric modeling, as estimates based solely on epistemic uncertainty systematically underestimate the variance of residual errors [10]. Our experiments indicate that the same shortcoming exists in rating prediction. On the MovieLens-1M, MovieLens-100K, and Amazon Digital Music datasets, the standard deviations of predictions generated by MC dropout, deep ensemble models, and single-head backpropagation Bayesian models are one order of magnitude smaller than the actual error, resulting in NLL values that are two to four orders of magnitude higher than those of any method that simultaneously models single-sample noise. Therefore, heteroscedastic aleatoric modeling is not an optional optimization measure but a necessary component for calibrating collaborative filtering; moreover, there has been no systematic comparative study to date between epistemic-only designs and heteroscedastic designs in this context.

In this paper, we propose BayesNCF-v2, a single-model-based heteroscedastic Bayesian neural collaborative filtering framework. The model employs deterministic user and item embeddings, a MLP feature extractor, and a Bayes-by-Backprop output layer. Building on this foundation, we add a heteroscedastic variance head trained via a Gaussian negative log-likelihood (NLL) objective function. Consequently, its uncertainty is decomposed into aleatoric and epistemic components. In this paper, we conduct a comparative evaluation of BayesNCF-v2 against standard NCF, MC dropout, deep ensemble, a heteroscedastic MC dropout variant, a heteroscedastic deep ensemble, BayesNCF-v1, and a probabilistic matrix factorization (PMF) baseline. Our main contributions are as follows:

1. We propose BayesNCF-v2, a single-model heteroscedastic Bayesian NCF, and demonstrate that, compared to standard NCF, it significantly reduces ECE and NLL on three public datasets. Furthermore, its calibration results show no statistically significant difference from those of heteroscedastic MC dropout, while using only about one-fifth of the parameters.
2. A systematic calibration comparison was conducted between methods based solely on epistemic and those based on heteroscedastic uncertainty. The results show that designs based solely on epistemic underestimate the prediction variance by one order of magnitude, leading to NLL performance that is two to four orders of magnitude worse.
3. We demonstrate that the learned aleatoric variance adapts to data sparsity: for cold-start users, this variance increases, thereby reducing the ECE for cold-start users across the three datasets. Furthermore, this calibration advantage persists even when future interactions are excluded from training via temporal segmentation.
4. We provide complementary calibration diagnostics, including predictive-interval coverage and PIT tests, ECE bin- and MC-sample sensitivity, a KL-weight/prior-scale sensitivity analysis, a Bayesian feature-layer variant, and a joint user × item cold-start stratification. The results demonstrate that the calibration advantage of BayesNCF-v2 is robust to these design choices.

The remainder of this paper is organized as follows. Section 2 reviews relevant research on Bayesian networks, uncertainty calibration, and uncertainty in recommendation systems. Section 3 describes the BayesNCF-v2 architecture and its training objectives. Section 4 details the experimental setup, while Section 5 presents key comparative analyses, time robustness, and hierarchical cold-start analysis. Section 6 summarizes this paper.

## 2. Related Work

### 2.1. Bayesian Neural Networks

Bayesian neural networks (BNNs) assign a probability distribution to the weights rather than estimating them from training data, and train the weights by minimizing the variational free energy. Blundell et al. proposed Bayes by Backprop, a variance-reduction scheme that estimates the ELBO gradient using samples from the variational posterior distribution [3]. Kingma et al. introduced a local reparameterization technique that samples layer activation values rather than weights, thereby further reducing the gradient variance [11]. Gal and Ghahramani proved that applying dropout during the testing phase can approximate Bayesian inference in deep neural networks [4]. Lakshminarayanan et al. proposed deep ensemble models, which estimate prediction uncertainty through the diversity of models trained independently based on appropriate scoring rules [5]. These three categories of methods serve as the standard baselines for uncertainty assessment in neural network models, and this paper will adopt all of them.

### 2.2. Uncertainty Calibration

Calibration measures whether a model’s confidence matches its empirical accuracy. For classification, Guo et al. showed that modern networks are miscalibrated and proposed temperature scaling as a post hoc fix [12]. For regression, Kuleshov et al. formalized calibration through quantile coverage and proposed a PIT-based diagnostic [13], while Lakshminarayanan et al. argued that proper scoring rules such as the negative log-likelihood (NLL) encourage calibrated predictive distributions without post hoc adjustment [5]. Kendall and Gal decomposed total predictive uncertainty into an aleatoric part and an epistemic part, and showed in computer vision that a heteroscedastic output head trained with a Gaussian NLL is necessary for well-calibrated regression predictions, because epistemic-only estimates systematically understate the residual variance [10]. In this paper we adopt a regression ECE that bins test samples by their predicted standard deviation and compares each bin’s RMSE with its mean predicted standard deviation, following the regression-calibration literature [13]. Uncertainty quantification is also central to high-dimensional statistical inference, where inferential reliability under sparse frequentist and Bayesian models is an active topic [14,15].

### 2.3. Uncertainty in Recommender Systems

In the field of collaborative filtering, probabilistic modeling encompasses methods ranging from Bayesian probability matrix factorization [16] to variational autoencoders for implicit feedback [17]. Recently, researchers have proposed deep recommendation systems capable of handling uncertainty. The BDCF utilizes Bayesian deep learning for uncertainty-aware ranking [6]. The BDECF combines Bayesian networks with ensemble learning and attention mechanisms for collaborative filtering [7]. Ref. [8] introduces a balance between exploration and exploitation via variational autoencoders. And the Wasserstein dependency graph Attention Network uses probabilistic embeddings to model uncertainty [9]. A common feature of these methods is that uncertainty is represented solely through model parameters or embeddings—that is, through epistemic components. While the observation noise in the ratings themselves is either ignored or assumed to be a fixed value.

### 2.4. Gap

In the field of rating prediction, the consequences of ignoring aleatoric noise have not yet been systematically studied. Our experiments show that on the MovieLens-1M, MovieLens-100K, and Amazon Digital Music datasets, predictions based solely on epistemic methods yield a standard deviation that is approximately one order of magnitude smaller than the actual error; their NLL values are two to four orders of magnitude worse than those of any heteroscedastic method; their regression ECE values range from 0.75 to 1.08. Therefore, this paper compares purely epistemic and heteroscedastic designs under a unified framework and proposes BayesNCF-v2, a single-model heteroscedastic Bayesian NCF that achieves calibration performance comparable to ensemble models at minimal computational cost.

## 3. Proposed Method

**Figure 1:**
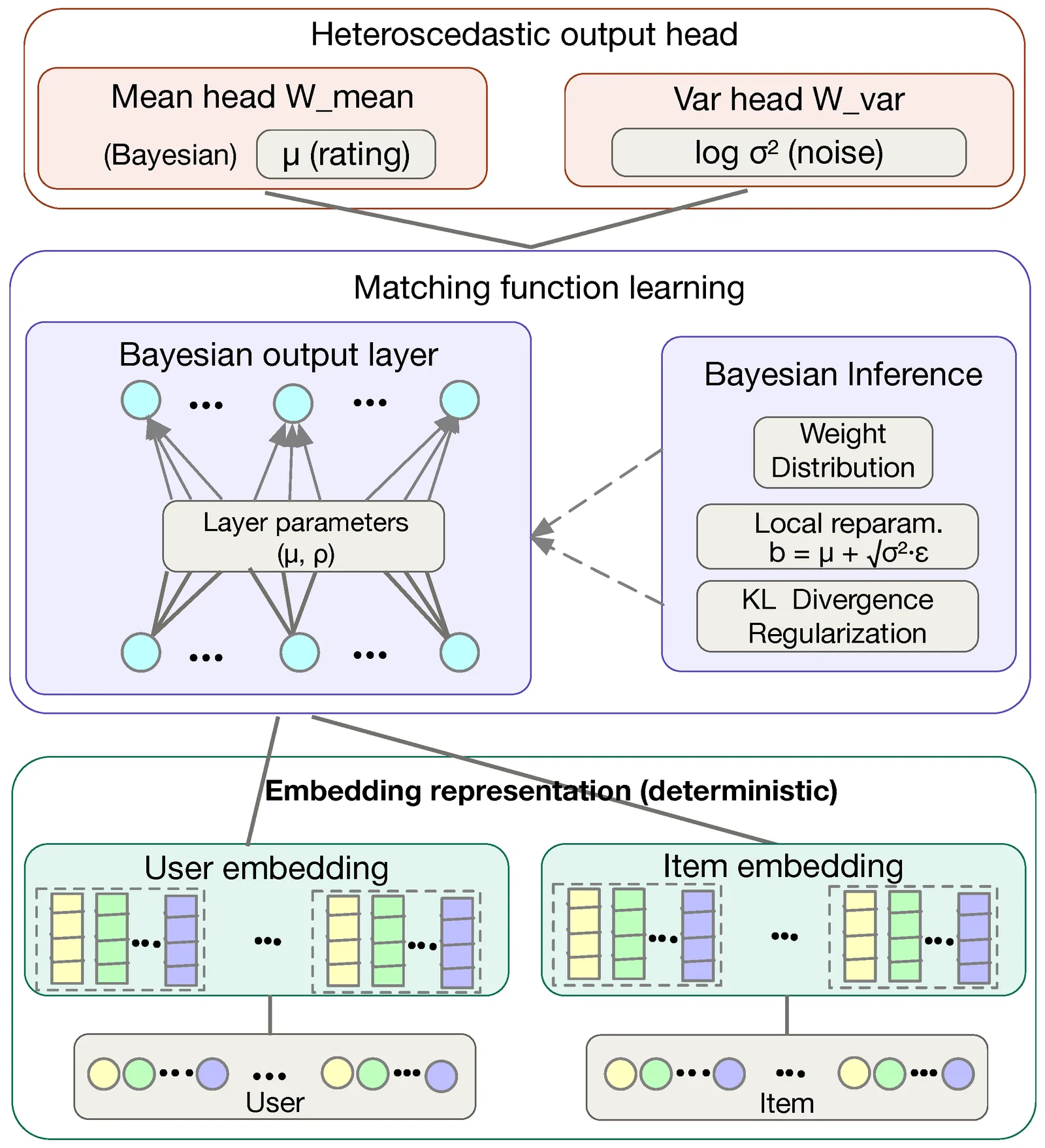
Architecture of BayesNCF-v2. Deterministic user and item embeddings are concatenated and passed through an MLP feature extractor. A Bayesian linear output layer predicts the rating mean on the raw [1,5] scale, and a separate variance head predicts the log-variance for heteroscedastic aleatoric uncertainty.

We propose BayesNCF-v2, which consists of deterministic user and item embeddings, a deterministic MLP feature extractor, a Bayesian output layer for predicting the mean score, and a deterministic heteroscedasticity head for predicting the logarithmic variance of each sample. The architecture of BayesNCF-v2 is shown in Figure 1.

### 3.1. Problem Formulation

Given observed user–item ratings D={(u,i,r)∣u∈U,i∈I,r∈[1,5]}, the goal is to learn f:U×I→R predicting the rating of user *u* for item *i*. In standard NCF [1], *f* is parameterized by point-estimate weights w and trained by minimizing the MSE. We extend this in two ways: the final prediction layer carries a variational posterior over its weights, and the model additionally predicts the per-sample observation noise. All metrics are computed directly on the raw 1–5 rating scale.

### 3.2. Architecture

User and item embeddings are deterministic tables $e_{u},e_{i}\in R^{d}$, initialized with small Gaussian noise N(0,0.01). The concatenation $\left[e_{u};e_{i}\right]$ is passed through a two-layer MLP with ReLU activations and dropout, producing a feature vector $h\in R^{d_{h}}$.

#### 3.2.1. Bayesian Output Layer

The rating mean is produced by a single Bayesian linear layer. Its weight posterior is

(1) $$q\left(w|D\right)=N\left(w|\mu ,\sigma^{2}\right),\sigma =\mathrm{softplus}\left(\rho \right).$$

During training, weights are sampled via the reparameterization

(2) w=μ+σ⊙ε,ε∼N(0,I).

During evaluation, the posterior mean μ is used for point prediction. The raw output is mapped to the rating scale through

(3) $$r=1+4\;\mathrm{sigmoid}(w^{⊤}h+b),$$

which keeps every prediction inside [1,5] and removes the normalization–denormalization round trip of earlier pipelines. Importantly, the epistemic component reflects only the variance of the Bayesian output layer’s weight posterior; the embeddings and representation layers are deterministic, so uncertainty in the learned user representation is not included.

#### 3.2.2. Heteroscedastic Variance Head

A deterministic two-layer head maps h to $\log\sigma_{\mathrm{al}}^{2}$, clamped to [−7,2] for numerical stability. This head predicts the observation noise of each user–item pair, and is the structural component that distinguishes BayesNCF-v2 from BayesNCF-v1, the ablation with this head removed. In our experiments the clamp is inactive at the optimum: on the MovieLens-100K test set, the learned log-variances lie in ≈[−0.93,+0.03] (0% of samples at either bound), so the clip only stabilizes training and never constrains the predictive mean.

### 3.3. Training Objective

We assume a heteroscedastic Gaussian likelihood

(4) $$p\left(r|u,i,w\right)=N(r|\mu_{w}\left(u,i\right),\sigma_{w}^{2}\left(u,i\right)).$$

The loss for one observation is

(5) $$L_{\mathrm{NLL}}=\frac{1}{2}\left(\frac{{\left(r-\mu \right)}^{2}}{\sigma^{2}}+\log\sigma^{2}\right),$$

whose first term is a precision-weighted squared error and whose second term prevents the model from trivially inflating $\sigma^{2}$.

The complexity cost is the closed-form KL divergence between the Gaussian posterior of the Bayesian layer and the prior $p\left(w\right)=N\left(0,\sigma_{p}^{2}I\right)$:

(6) $$\mathrm{KL}\left[q∥p\right]=\sum_{j}\left[\log\frac{\sigma_{p}}{\sigma_{j}}+\frac{\sigma_{j}^{2}+\mu_{j}^{2}}{2\sigma_{p}^{2}}-\frac{1}{2}\right].$$

The total mini-batch objective is

(7) $$L=\frac{1}{\left|B\right|}\sum_{(u,i,r)\in B}L_{\mathrm{NLL}}+\lambda \;\mathrm{KL}\left[q∥p\right],$$

with $\lambda =10^{-3}$. The Gaussian is used as a working likelihood for tractability and to align with the NLL/ECE evaluation. Because the predictive mean is constrained to [1,5] by the sigmoid output, the predictive distribution never leaves the valid rating range even though the Gaussian tails extend beyond it. Ordinal, beta, and censored-Gaussian likelihoods are discussed as future work; the over-coverage observed on the sparse Amazon subset (Section 5) is a direct consequence of the Gaussian assumption on concentrated discrete ratings. We additionally evaluate the sensitivity of the results to the KL weight $\lambda \in \{10^{-4},10^{-3},10^{-2}\}$ and the prior scale $\sigma_{p}\in \left\{0.1,0.3,1.0\right\}$ on MovieLens-100K (Section 5 ); the regression ECE stays within 0.035–0.060 across the grid, below the NCF baseline (0.074). We therefore interpret all predictive intervals and coverage diagnostics as approximations induced by this working likelihood, not as exact statements about the discrete rating distribution. Following the posterior-collapse literature, we initialize μ∼N(0,0.05) and ρ=−2.5, and use a prior $\sigma_{p}=0.3$; these choices keep the variational posterior learnable instead of pinned near a tight initialization. Parameters are optimized with Adam [18] and gradient clipping, with early stopping on the validation RMSE. Algorithm 1 summarizes the procedure.
**Algorithm 1** BayesNCF-v2 training**Require:** 
Training data D, prior $\sigma_{p}$, KL weight λ, epochs *E*, patience *P*  1:Initialize μ∼N(0,0.05), ρ=−2.5, biases =0  2:**for** epoch =1 to *E* **do**  3:     **for** each mini-batch B **do**  4:         Sample ε∼N(0,I)  5:         σ←softplus(ρ); w←μ+σ⊙ε  6:         $\mu ,\log\sigma^{2}\leftarrow \mathrm{forward}\left(u,i,w\right)$  7:         $L\leftarrow L_{\mathrm{NLL}}+\lambda \;\mathrm{KL}\left[q∥p\right]$  8:         Update θ={μ,ρ} via Adam with gradient clipping  9:     **end for**10:     Early stop if the validation RMSE has not improved for *P* epochs11:**end for**12:**return** Learned parameters $\theta^{*}$

### 3.4. Uncertainty Decomposition at Inference

At test time, T=30 stochastic forward passes sample weights from the posterior. Following Kendall and Gal [10], the total predictive variance is decomposed as

(8) $$\mathrm{Aleatoric}=\frac{1}{T}\sum_{t=1}^{T}\sigma_{t}^{2},\;\;\;\;\;\;\;\mathrm{Epistemic}=\frac{1}{T}\sum_{t=1}^{T}{\left(\mu_{t}-\bar{\mu }\right)}^{2},$$

with $\bar{\mu }=\frac{1}{T}\sum_{t}\mu_{t}$.

(9) Total=Aleatoric+Epistemic

More precisely, the predictive distribution produced by BayesNCF-v2 is the Gaussian mixture

(10) $$q\left(r|x\right)=\frac{1}{T}\sum_{t=1}^{T}N\;\left(r\;|\;\mu_{t},\;{\hat{\sigma }}^{2}\left(x\right)\right),\;\;\;\;\;\;\mu_{t}=g\left(w_{t}^{⊤}h+b\right),\;\;\;\;\;g\left(z\right)=1+4\;\mathrm{sigmoid}\left(z\right),$$

where ${\hat{\sigma }}^{2}\left(x\right)=\exp\left(\mathrm{lv}\left(x\right)\right)$ is the variance-head output, a deterministic function of the input features that is shared across posterior draws. By the law of total variance, the variance of this mixture is exactly

(11) $${\mathrm{Var}}_{r∼q}\left[\;r|x\;\right]=\underset{\mathrm{Aleatoric}}{\underbrace{{\hat{\sigma }}^{2}\left(x\right)}}+\underset{\mathrm{Epistemic}}{\underbrace{\frac{1}{T}\sum_{t=1}^{T}{\left(\mu_{t}-\bar{\mu }\right)}^{2}}},$$

so the reported decomposition is exact for the predictive distribution that the model actually defines; it does not require propagating a Gaussian distribution through *g*. The nonlinear mapping enters only through the component means: because *g* is monotone, the components remain ordered on the rating scale, and the compression of large raw deviations is reflected exactly in the empirical between-component variance. (For small deviations, the delta method gives $\mathrm{Var}\left[g\left(Z\right)\right]\approx {\left[g^{\prime }\left(EZ\right)\right]}^{2}\;\mathrm{Var}\left[Z\right]$ with $g^{\prime }\left(z\right)=4\;\mathrm{sigmoid}\left(z\right)\left(1-\mathrm{sigmoid}\left(z\right)\right)\leq 1$, which provides intuition for the compression but is not needed for the identity above.) Two remarks complete the justification. (i) Because ${\hat{\sigma }}^{2}$ is shared across draws, the Gaussian NLL fits it to the average squared residual of the sampled means; when the epistemic variance is small, this coincides with the per-component noise to within the epistemic share. In our experiments the epistemic share is only about 1–2% of the total predictive variance, so the shared-variance assumption changes the predictive variance by at most the epistemic share and the predictive standard deviation by under one percent; the coverage diagnostics confirm that the resulting intervals are not systematically mis-sized. (ii) Exactness with a large epistemic share would require conditioning the variance head on the drawn weights, as in per-sample dropout implementations; we leave this for future work. MC dropout, deep ensembles, and BayesNCF-v1 can only estimate the epistemic term.

### 3.5. Comparison with Alternative Approaches

Table 1 summarizes the differences between BayesNCF-v2 and the baselines. Because only the final output layer is Bayesian, the parameter count is only about 1.04× that of plain NCF, in contrast to the 5× cost of a five-member ensemble.

## 4. Experimental Setup

### 4.1. Datasets

We evaluate on three classic datasets:

- MovieLens-1M [19]: 1,000,209 ratings, 6040 users, and 3706 items.
- MovieLens-100K [19]: 100,000 ratings, 943 users, and 1682 items.
- Amazon Digital Music [20]: A sparse subset with 130,434 ratings, 100,952 users, and 70,519 items.

All ratings are used on the raw 1–5 scale. Each dataset uses (i) a random 80/20 split and (ii) a temporal split in which the most recent 20% of interactions form the test set. Under the temporal split, interactions are sorted by timestamp; users and items that appear only in the test period are cold-start entities whose ratings are predicted from the trained embeddings, and no test-period interaction is used in training. All methods share the same splits and the same five model seeds range from 2024 to 2028; results are reported as the mean ± std over seeds, and differences are tested with paired *t*-tests on the per-seed values.

### 4.2. Baselines

All methods share the same embeddings, the same two-hidden-layer MLP, the same optimizer, batch size of 512, and early-stopping patience of 10:

- NCF: Standard NCF with point-estimate weights, trained with the MSE; no uncertainty output.
- MC dropout: NCF with dropout retained at test time; uncertainty from 30 stochastic forward passes [4].
- DeepEnsemble: Five independently trained NCFs [5].
- BayesNCF-v1: BayesNCF-v2 without the heteroscedastic variance head; epistemic uncertainty only.
- Heteroscedastic MC dropout: MC dropout with a per-sample variance head trained with the Gaussian NLL.
- Heteroscedastic deep ensemble: Five members, each with its own variance head, trained with the Gaussian NLL.
- PMF: Probabilistic matrix factorization [16], trained under the same five-seed protocol with a fixed $\sigma^{2}$ fitted on the held-out calibration slice.
- BayesNCF-v2 (Ours): Single-model heteroscedastic Bayesian NCF.

### 4.3. Evaluation Metrics

Rating accuracy is measured by the RMSE and MAE on the raw 1–5 scale. Calibration is measured by the Gaussian NLL and by a regression ECE that bins test samples into ten quantiles by predicted standard deviation and computes by

(12) $$\sum_{m}|B_{m}|/N\cdot |\mathrm{RMSE}\left(B_{m}\right)-\bar{\sigma }\left(B_{m}\right)|.$$

Ranking is measured by Recall@10 and NDCG@10 over 100 candidates per test rating. For the point-estimate NCF, NLL and ECE are computed with a single fixed $\sigma^{2}$ fitted on a held-out 10% calibration slice drawn from the tail of the training partition; this slice is never used for testing. For every paired comparison we report the mean difference, its 95% confidence interval, Cohen’s $d_{z}$, and exact *p*-values from paired *t*-tests and permutation tests; because five seeds bound the number of independent samples, we discuss the multiple-comparison issue when interpreting the pairwise differences.

## 5. Results and Discussion

### 5.1. Main Results

**Figure 2:**
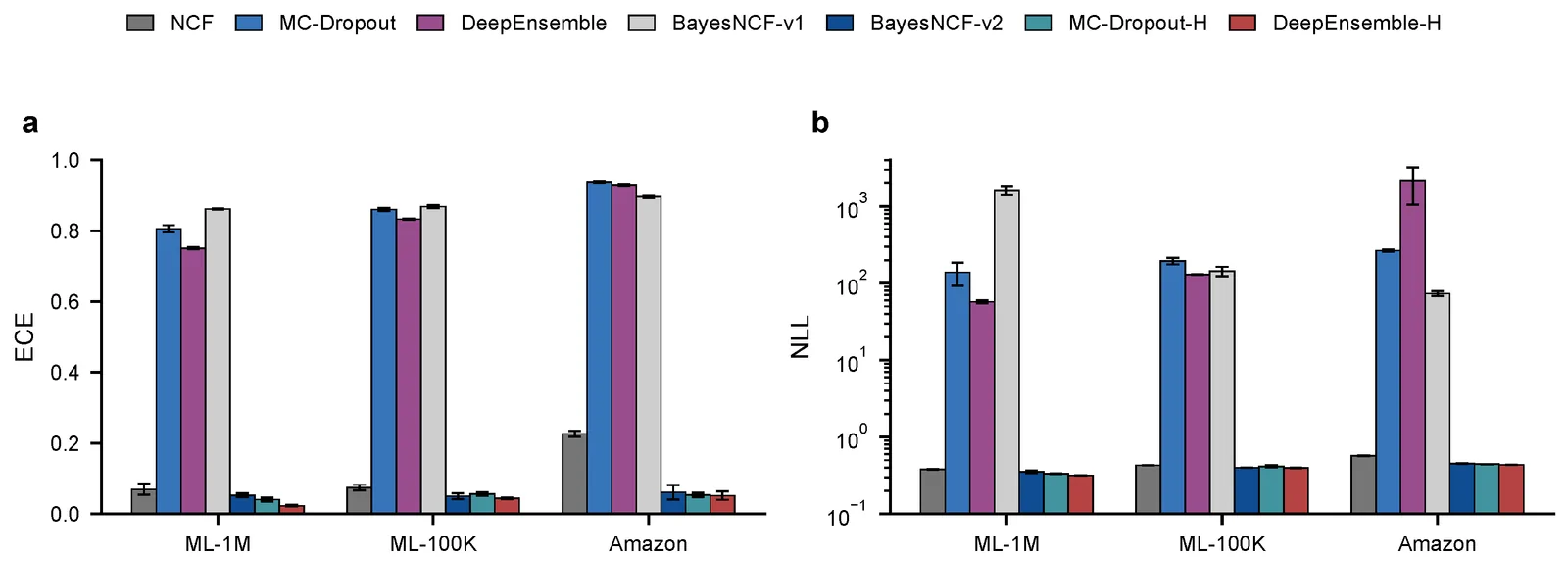
Main comparison on the random split: (**a**) regression ECE and (**b**) NLL per method on MovieLens-1M, MovieLens-100K, and Amazon Digital Music. Error bars are one standard deviation over five seeds.

Figure 2 and Table 2, Table 3 and Table 4 show the main comparison on the random split. Three findings hold consistently across all three datasets.

First, epistemic-only methods are severely miscalibrated. MC dropout, deep ensembles, and BayesNCF-v1 produce predictive standard deviations roughly an order of magnitude smaller than the realized error; their NLL is two to four orders of magnitude worse than any heteroscedastic method, and their regression ECE reaches 0.75–1.08. This failure is expected from the regression-calibration literature [10,13], but it is rarely quantified for recommender systems, and it shows that aleatoric modeling is not an optional refinement in rating prediction.

Second, BayesNCF-v2 significantly improves calibration over the point-estimate NCF baseline. ECE is reduced by 25–73%, and NLL is reduced by 7–20%, with generally comparable predictive accuracy (RMSE within 0.8% across settings; e.g., +0.8% on Amazon under the random split). The largest calibration gain occurs on the sparsest dataset, Amazon Digital Music.

Third, the calibration of BayesNCF-v2 is statistically indistinguishable from heteroscedastic MC dropout on all six settings, and close to a five-member heteroscedastic deep ensemble: ECE differences versus the ensemble are not significant on four of six settings, with only MovieLens-1M showing a significant gap, at roughly one-fifth of the ensemble’s parameters. Heteroscedastic ensembles remain the strongest calibration method; BayesNCF-v2 attains ensemble-level calibration with a single model.

Fourth, the PMF baseline is consistently worse calibrated than BayesNCF-v2: its ECE is 0.118, 0.263, and 0.906 on MovieLens-1M, MovieLens-100K, and Amazon, versus 0.052, 0.050, and 0.061 for BayesNCF-v2. On the sparse Amazon subset the fixed-σ protocol is unfavorable to PMF—its calibration-slice variance is far below the realized RMSE, which further motivates per-sample variance estimation.

Finally, to separate the effect of the data from that of the parameterization on the small epistemic share, we trained a variant with a Bayesian first feature layer on MovieLens-100K (random split, three seeds). The epistemic share of the predictive variance increases from 1.9% (v2) to 5.2%, the feature-layer posterior standard deviation reaches ≈ 0.30, and calibration remains in the same low range at a modest RMSE cost. The small epistemic share in v2 therefore reflects the parameterization as well as the data, and making feature layers Bayesian does not change the qualitative conclusion that aleatoric variance dominates.

### 5.2. Component Analysis

Table 5 isolates the two structural components of BayesNCF-v2 on MovieLens-1M (random split, five seeds): the Bayesian output layer with its KL term, and the heteroscedastic variance head. Comparing BayesNCF-v1 with BayesNCF-v2 keeps the Bayesian layer and the KL term fixed and adds only the variance head: ECE drops from 0.861 to 0.052. Comparing heteroscedastic MC dropout with BayesNCF-v2 keeps the variance head and the Gaussian NLL and adds only the Bayesian layer and its KL term: ECE changes from 0.041 to 0.052, a difference whose 95% confidence interval includes zero and which is not statistically significant. The variance modeling therefore appears to be the main contributor to the calibration improvement observed in these experiments.

Two caveats apply to this attribution. First, the comparison NCF versus BayesNCF-v1 changes the Bayesian layer and the KL regularization jointly, so the effect of KL regularization alone cannot be isolated with the current design: removing the KL term from the Bayesian output layer would reduce the objective to a deterministic MAP estimate and abandon the variational formulation, so the KL term is intrinsic to the Bayes-by-Backprop layer rather than a free hyperparameter of a deterministic network. Second, because the KL term is the only loss difference between the Bayesian and non-Bayesian variants that share the same architecture and variance head, the Bayesian-versus-KL attribution is entangled by construction; we report the joint contrast and do not claim that either component alone drives the results.

### 5.3. Robustness Under the Temporal Split

Table 6 repeats the comparison when future interactions are excluded from training. The conclusions are unchanged: ECE of BayesNCF-v2 versus NCF is reduced by 60% on MovieLens-1M, 29% on MovieLens-100K, and 73% on Amazon (all significant), while heteroscedastic baselines remain on par.

### 5.4. Cold-Start Stratification

**Figure 3:**
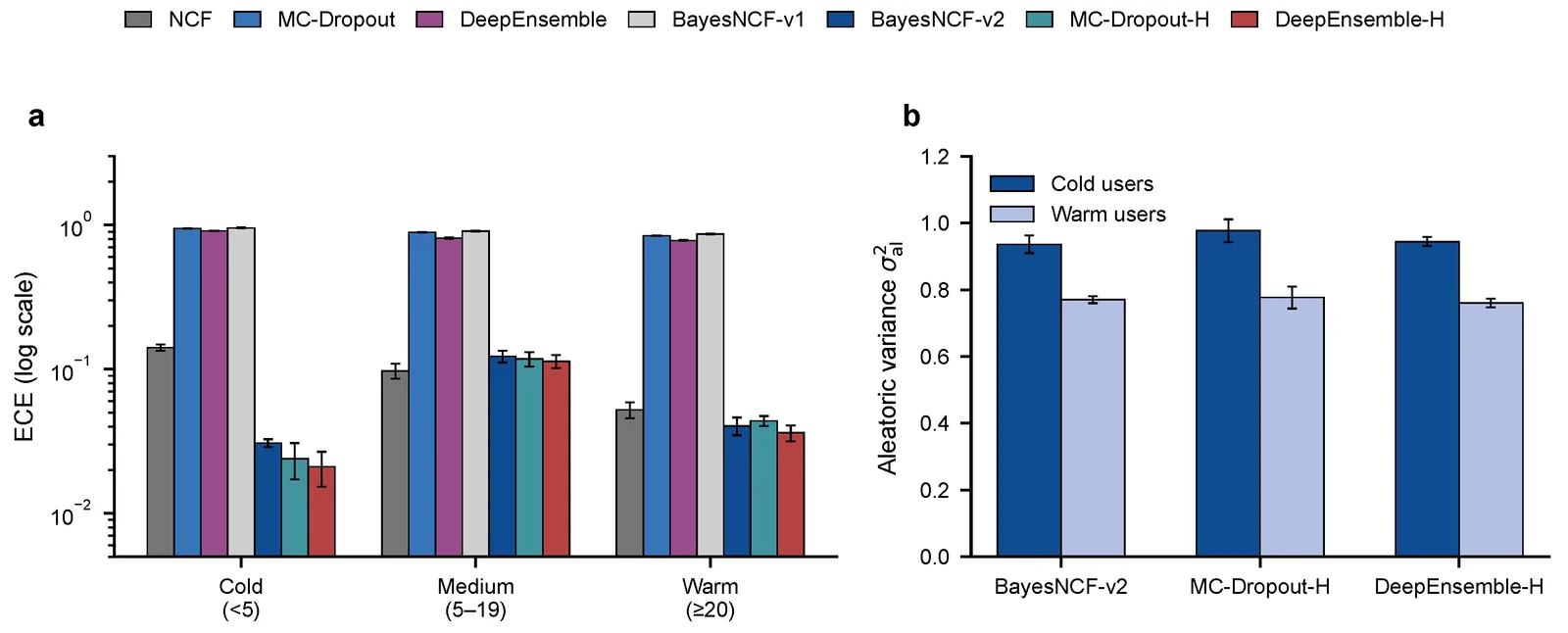
Stratified evaluation (temporal split, 5 seeds): (**a**) regression ECE by user bucket (cold/medium/warm, log scale); (**b**) learned aleatoric variance for cold versus warm users. Error bars are one standard deviation over five seeds.

Under the temporal split, users are stratified by their training interaction count. Figure 3 shows the user-level results. BayesNCF-v2 reduces cold-user ECE by 78% on MovieLens-1M, by 30% on MovieLens-100K, and by 72% on Amazon. The learned aleatoric variance increases for cold-start users, which is the mechanism behind the improved per-sample calibration. The epistemic fraction remains small and does not increase for cold users, so the improvement is driven by the aleatoric head, not by posterior uncertainty.

We additionally stratified by item interaction counts and by the joint user × item cells (Table 7). For cold items (temporal split), BayesNCF-v2 reduces ECE from 0.298 to 0.231 on MovieLens-1M and from 0.249 to 0.221 on MovieLens-100K. In the joint cold-user × cold-item cells the ECE is 0.303 vs. 0.463 (MovieLens-1M), 0.252 vs. 0.276 (MovieLens-100K), and 0.075 vs. 0.334 (Amazon). On MovieLens-1M the calibration gain holds in all four user × item cells; on MovieLens-100K and Amazon, the gains concentrate on cold-user cells, and the small warm-user × cold-item cells are noisy, which we discuss in the limitations.

### 5.5. Calibration Curves and Uncertainty Decomposition

**Figure 4:**
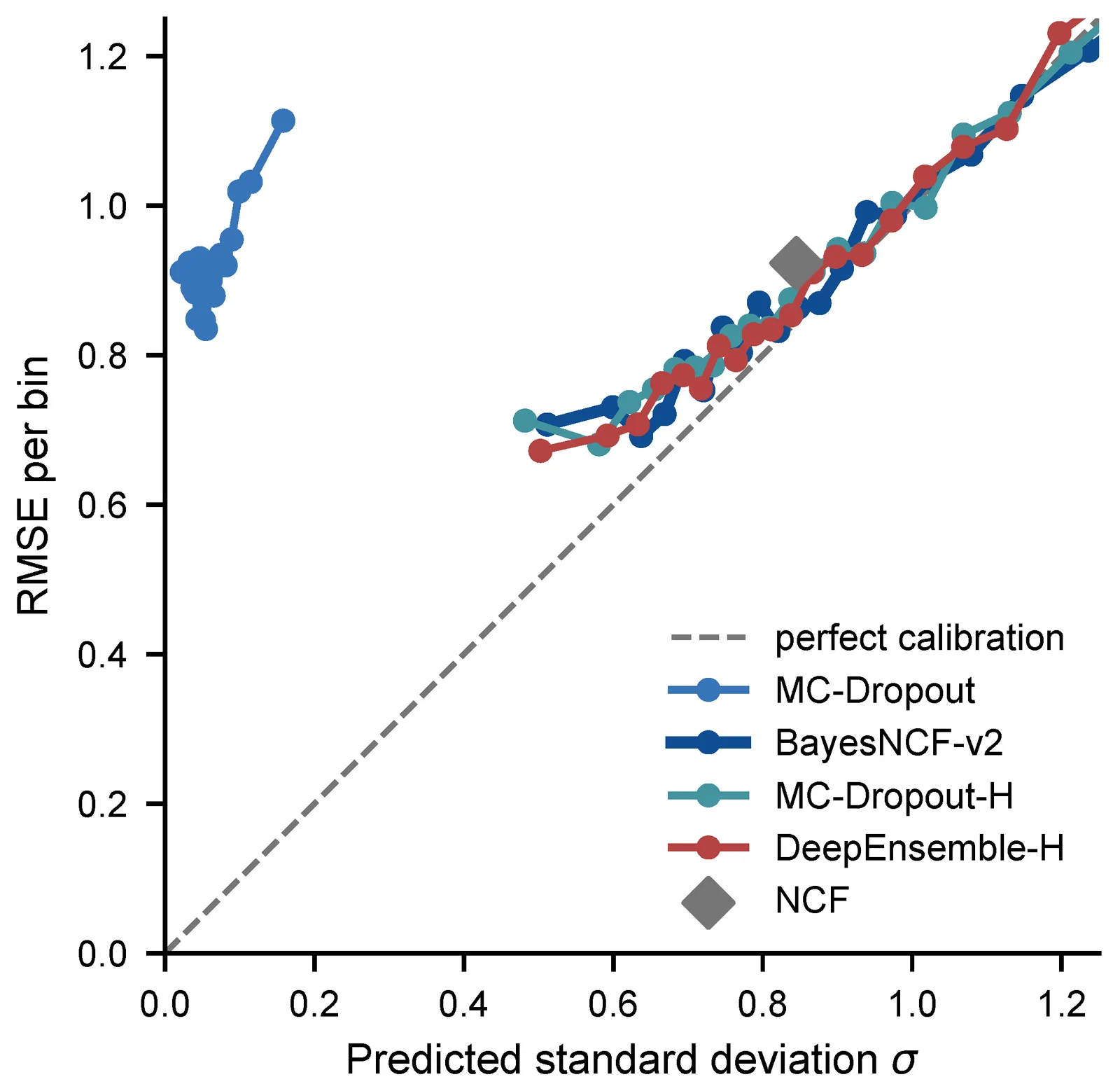
Calibration curves on MovieLens-100K (seed 2024): the per-bin RMSE versus the predicted standard deviation (20 quantile bins). The dashed diagonal is perfect calibration.

**Figure 5:**
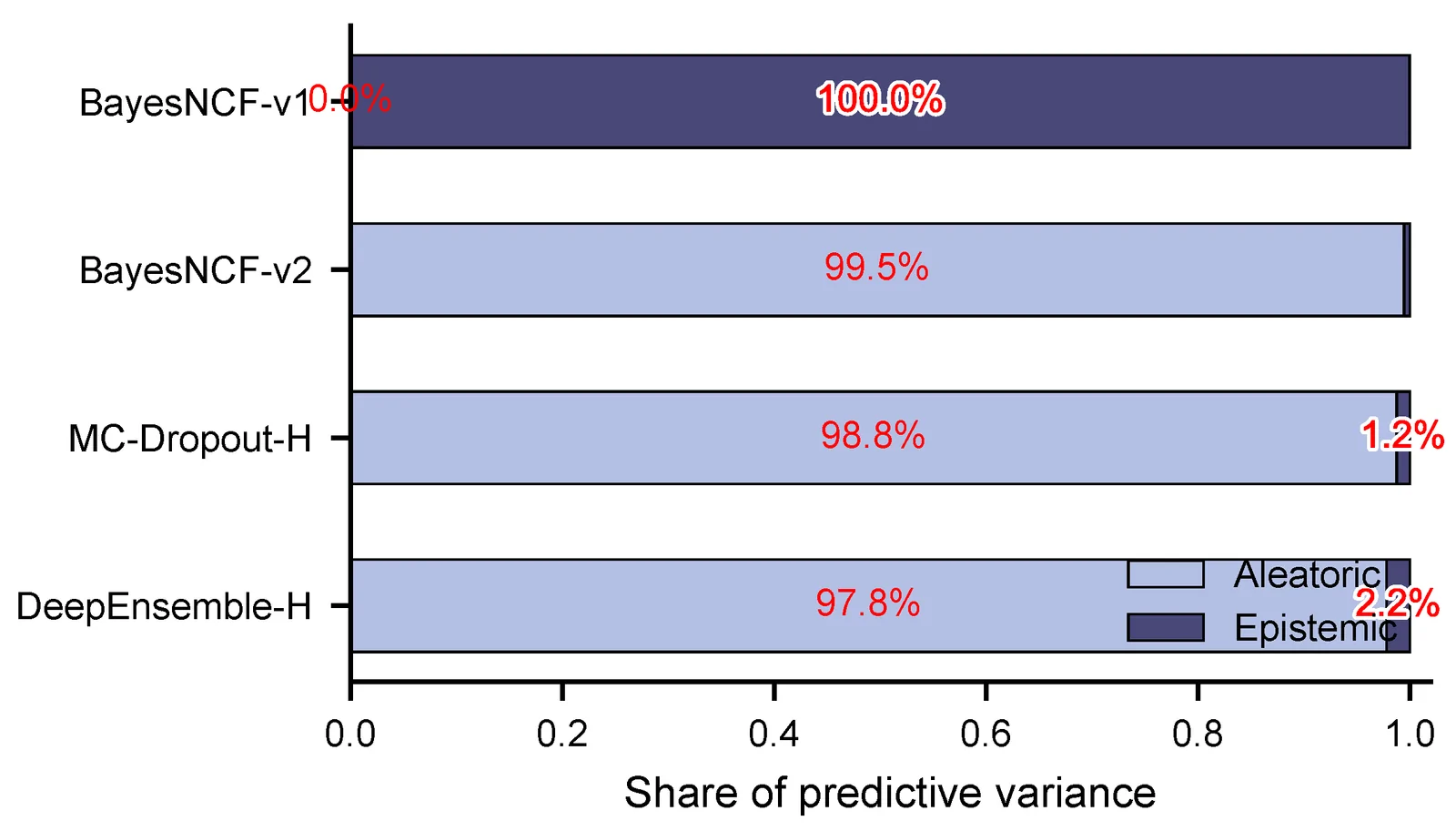
Epistemic versus aleatoric share of the total predictive variance on MovieLens-1M (5 seeds).

Figure 4 shows calibration curves on MovieLens-100K: heteroscedastic methods lie near the diagonal, while MC dropout collapses near the origin. Figure 5 shows that aleatoric variance accounts for 97.8–99.9% of the total predictive variance. The Bayesian layer’s posterior is learnable rather than collapsed: its standard deviation moves from the initialization value softplus(−2.5)≈0.079 to approximately 0.21 after training (KL ≈30). The learned aleatoric variance should be read as conditional predictive variability under the proposed model; it may also absorb preference heterogeneity, sparsity, and model misspecification, and is not a direct measure of intrinsic noise. The learned aleatoric variance should be read as conditional predictive variability under the proposed model; it may also absorb preference heterogeneity, sparsity, and model misspecification, and is not a direct measure of intrinsic noise. These numbers also delimit the practical contribution of the Bayesian component in this study. Because the epistemic share is only about 1–2% of the total predictive variance, the Bayesian layer does not materially change the predictive intervals, and BayesNCF-v2 is statistically indistinguishable from heteroscedastic MC dropout, which uses no Bayesian layer at all (Section 5.2). The Bayesian formulation instead provides a principled single-model posterior and the epistemic term that makes the uncertainty decomposition possible. For applications that require only calibrated predictive intervals, the heteroscedastic variance head is sufficient; for applications that require epistemic uncertainty, the posterior matters, and its share can grow under distribution shift or when feature layers are made Bayesian (Section 5.1).

### 5.6. Calibration Diagnostics

**Figure 6:**
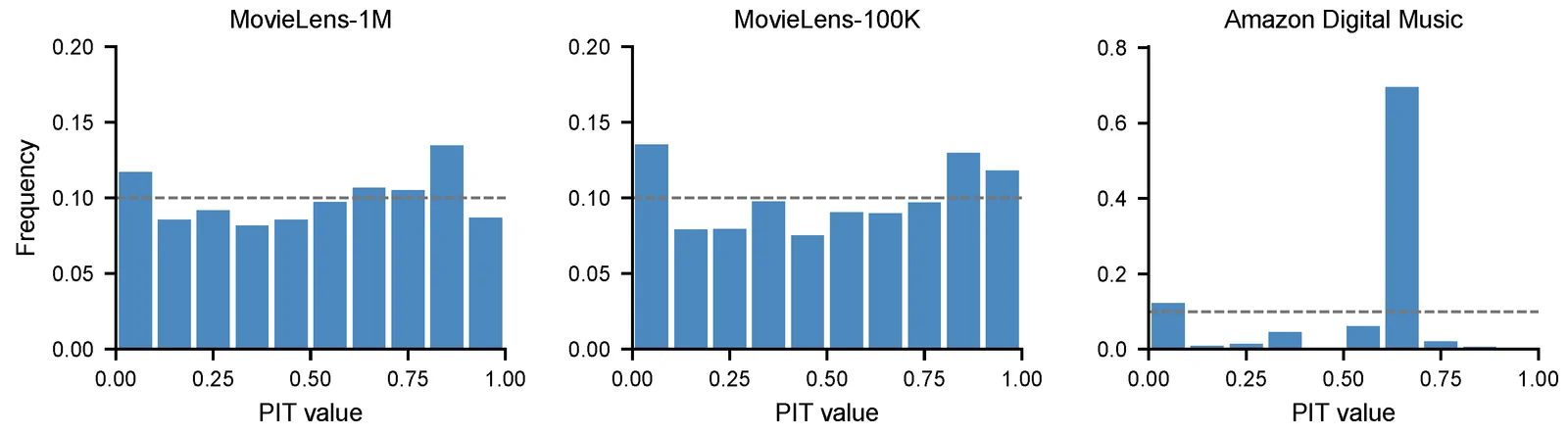
PIT histograms of BayesNCF-v2 on the temporal test sets (representative seed 2024; seed-averaged statistics in Table 8): MovieLens-1M, MovieLens-100K, and Amazon Digital Music. The dashed line is the uniform reference.

Table 8 reports predictive-interval coverage at nominal levels 50/80/90/95% together with the PIT-based Kolmogorov–Smirnov (KS) statistic for BayesNCF-v2 on the temporal test sets, aggregated over all five model seeds (mean ± std). On the MovieLens datasets the empirical coverage is close to nominal (within 3–6 percentage points at the 50% level) and the PIT histograms are near-uniform (Figure 6, shown for a representative seed). On the sparse Amazon subset the intervals over-cover at the 50% level; as discussed in Section 3.3, this is a direct consequence of the Gaussian working likelihood on concentrated, discrete ratings, and the same pattern holds for every seed. Because ratings are discrete and bounded, we interpret these diagnostics cautiously: the Gaussian is a working likelihood, and near-nominal coverage on the MovieLens datasets does not imply that it exactly describes the rating distribution. Under the random split the Amazon values are similar.

Aggregated over the five seeds, the regression ECE is insensitive to the number of bins (10/15/20 bins: 0.040/0.039/0.040 on MovieLens-1M; 0.090/0.090/0.091 on MovieLens-100K; 0.090/0.090/0.091 on Amazon) and to the number of MC samples (T from 5 to 30 changes ECE by at most 0.003 on every dataset), supporting the choice of ten quantile bins and T = 30 throughout this paper. Table 9 summarizes the sensitivity analysis over the KL weight and the prior scale described in Section 3.3; the calibration results are stable across the whole grid.

### 5.7. Efficiency

BayesNCF-v2 uses 210K parameters on the MovieLens datasets versus 1047K for a five-member heteroscedastic ensemble, and a single forward pass at inference is comparable in cost to NCF; MC sampling for uncertainty estimation adds a *T*-fold inference cost for any method that uses it. Table 10 reports wall-clock timings measured on the CPU (batch size 512, MovieLens checkpoints). MC sampling adds a *T*-fold cost for methods that use it, while the five-member ensemble costs 5× the parameters and roughly 5× the point-inference time.

### 5.8. Limitations and Discussion

The results place limitations on this contribution in three respects. First, the heteroscedastic deep ensemble model achieves the best overall calibration performance, particularly on the MovieLens-1M dataset. BayesNCF-v2 aims to achieve ensemble-level calibration at a fraction of the cost, rather than outperforming ensemble models. Second, the cold-start advantage is most pronounced among cold users. For cold items and in buckets of marginally warm users within sparse Amazon subsets, single-sample calibration is less reliable. Third, in these scenarios, the epistemic component contributes negligibly to calibration; applications requiring decision-making based on epistemic uncertainty fall outside the scope of this evaluation. The common conclusion drawn from the six comparison scenarios is that heteroscedastic aleatoric modeling—rather than relying solely on Bayesian posterior distributions—is the main driver of calibration in our experiments. Finally, these conclusions are limited to the methods and datasets examined here; the failure of epistemic-only methods may depend on model specification and posterior approximation, and the joint user × item cells with very small sample sizes should be interpreted with caution.

## 6. Conclusions

We propose a single-model heteroscedastic Bayesian neural collaborative filtering framework, BayesNCF-v2, which can jointly estimate both aleatoric and epistemic uncertainty in rating predictions. Across five seeds, three datasets, and under both random and temporal partitioning scenarios, compared to the point-estimation NCF baseline that does not account for uncertainty, this method reduces regression ECE by 25% to 73% and NLL by 7–20%, with generally comparable predictive accuracy (RMSE within 0.8% across settings). Its calibration performance is statistically indistinguishable from that of heteroscedastic MC dropout methods, while requiring only about one-fifth of the parameters to approach the performance of heteroscedastic deep ensemble models. The calibration advantage is most pronounced for cold-start users—as data scarcity increases, the learned aleatoric variance for these users also increases.

However, methods based solely on epistemic uncertainty systematically underestimate the prediction variance in score regression, resulting in NLL values that differ by two to four orders of magnitude, with regression ECE values ranging from 0.75 to 1.08. Heteroscedastic aleatoric modeling—rather than relying solely on Bayesian posterior distributions—is the main driver of calibration in these experiments. This failure of epistemic-only methods was observed for the specific models and datasets studied and may depend on model specification and posterior approximation.

Future work includes deepening the Bayesian component for applications that genuinely need epistemic uncertainty, extending the framework to graph-based architectures such as LightGCN [21], evaluating implicit-feedback settings, and using the calibrated uncertainty estimates for active learning and online exploration in production recommender systems.

## Figures and Tables

**Table 1:** Methodological comparison of uncertainty methods.

Property	MC-Drop	Ensemble	v1	MC-Drop-H	v2 (Ours)
True prior p(w)	No	No	Yes	No	Yes
Learned aleatoric $\sigma^{2}$	No	No	No	Yes	Yes
Epistemic $\sigma^{2}$	Yes	Yes	Yes	Yes	Yes
Loss	MSE	MSE	MSE + KL	NLL	NLL + λKL
Params vs. NCF	1×	5×	∼1.04×	∼1.04×	∼1.04×

**Table 2:** MovieLens-1M performance comparison.

Method	RMSE	MAE	NLL	ECE	R@10	NDCG@10
NCF	0.8804 ± 0.0011	0.6911 ± 0.0015	0.3802 ± 0.0037	0.0696 ± 0.0157	0.2623	0.1392
MC dropout	0.8802 ± 0.0010	0.6910 ± 0.0015	138.7 ± 46.8	0.8056 ± 0.0102	0.2623	0.1392
DeepEnsemble	0.8720 ± 0.0005	0.6847 ± 0.0007	57.2 ± 2.7	0.7505 ± 0.0029	0.2642	0.1412
BayesNCF-v1	0.8791 ± 0.0013	0.6889 ± 0.0013	1590 ± 197	0.8614 ± 0.0012	0.2586	0.1366
BayesNCF-v2	0.8759 ± 0.0004	0.6853 ± 0.0009	0.3539 ± 0.0138	0.0524 ± 0.0057	0.2614	0.1360
MC-Drop-H	0.8761 ± 0.0006	0.6874 ± 0.0020	0.3343 ± 0.0027	0.0408 ± 0.0057	0.2611	0.1366
Ensemble-H	0.8682 ± 0.0002	0.6815 ± 0.0004	0.3166 ± 0.0017	0.0232 ± 0.0023	0.2717	0.1441
PMF	0.8928 ± 0.0006	0.7026 ± 0.0005	0.4088 ± 0.0008	0.1181 ± 0.0005	0.2984	0.1600

**Table 3:** MovieLens-100K performance comparison.

Method	RMSE	MAE	NLL	ECE	R@10	NDCG@10
NCF	0.9256 ± 0.0014	0.7262 ± 0.0021	0.4303 ± 0.0013	0.0744 ± 0.0078	0.1958	0.0899
MC dropout	0.9254 ± 0.0015	0.7262 ± 0.0020	194.5 ± 18.6	0.8601 ± 0.0041	0.1958	0.0899
DeepEnsemble	0.9209 ± 0.0003	0.7235 ± 0.0005	129.8 ± 1.9	0.8330 ± 0.0013	0.1959	0.0898
BayesNCF-v1	0.9259 ± 0.0004	0.7267 ± 0.0013	143.6 ± 20.3	0.8686 ± 0.0039	0.1936	0.0884
BayesNCF-v2	0.9245 ± 0.0012	0.7249 ± 0.0017	0.3999 ± 0.0031	0.0504 ± 0.0083	0.1900	0.0819
MC-Drop-H	0.9235 ± 0.0012	0.7248 ± 0.0012	0.4153 ± 0.0125	0.0562 ± 0.0053	0.1898	0.0819
Ensemble-H	0.9179 ± 0.0006	0.7207 ± 0.0008	0.3969 ± 0.0019	0.0438 ± 0.0020	0.1946	0.0848
PMF	0.9682 ± 0.0028	0.7686 ± 0.0023	0.5926 ± 0.0042	0.2625 ± 0.0024	0.2980	0.1587

**Table 4:** Amazon Digital Music performance comparison. The dataset is a sparse subset.

Method	RMSE	MAE	NLL	ECE	R@10	NDCG@10
NCF	0.9882 ± 0.0006	0.6684 ± 0.0043	0.5694 ± 0.0077	0.2263 ± 0.0082	0.2087	0.1256
MC dropout	0.9883 ± 0.0007	0.6696 ± 0.0043	265.9 ± 8.5	0.9361 ± 0.0016	0.2087	0.1256
DeepEnsemble	0.9862 ± 0.0002	0.6607 ± 0.0015	2123 ± 1067	0.9277 ± 0.0021	0.2129	0.1305
BayesNCF-v1	0.9905 ± 0.0007	0.6551 ± 0.0059	73.2 ± 5.2	0.8961 ± 0.0028	0.2089	0.1270
BayesNCF-v2	0.9958 ± 0.0017	0.6640 ± 0.0144	0.4535 ± 0.0052	0.0610 ± 0.0209	0.2370	0.1454
MC-Drop-H	0.9921 ± 0.0007	0.6665 ± 0.0090	0.4441 ± 0.0018	0.0533 ± 0.0062	0.2349	0.1433
Ensemble-H	0.9895 ± 0.0005	0.6682 ± 0.0028	0.4375 ± 0.0020	0.0516 ± 0.0117	0.2378	0.1451
PMF	1.1304 ± 0.0005	0.9788 ± 0.0005	11.20 ± 0.22	0.9060 ± 0.0020	0.1686	0.1028

**Table 5:** Component ablation on MovieLens-1M (random split, five seeds).

Design	Bayesian Layer	Variance Head	ECE
NCF	No	No	0.0696 ± 0.0157
BayesNCF-v1	Yes	No	0.8614 ± 0.0012
MC-Dropout-H	No	Yes	0.0408 ± 0.0057
BayesNCF-v2	Yes	Yes	0.0524 ± 0.0057

**Table 6:** Temporal-split comparison.

Dataset	Method	RMSE	NLL	ECE	R@10	NDCG@10
ML-1M	NCF	0.9466 ± 0.0007	0.4582 ± 0.0028	0.0983 ± 0.0071	0.2297	0.1168
ML-1M	BayesNCF-v2	0.9473 ± 0.0009	0.4335 ± 0.0037	0.0393 ± 0.0033	0.2254	0.1136
ML-1M	MC-Drop-H	0.9462 ± 0.0004	0.4315 ± 0.0014	0.0372 ± 0.0045	0.2238	0.1135
ML-1M	Ensemble-H	0.9418 ± 0.0004	0.4241 ± 0.0023	0.0317 ± 0.0060	0.2260	0.1147
ML-100K	NCF	1.0259 ± 0.0011	0.5452 ± 0.0065	0.1271 ± 0.0156	0.2490	0.1271
ML-100K	BayesNCF-v2	1.0266 ± 0.0015	0.5330 ± 0.0071	0.0903 ± 0.0169	0.2220	0.1085
ML-100K	MC-Drop-H	1.0258 ± 0.0006	0.5302 ± 0.0059	0.0896 ± 0.0143	0.2341	0.1166
ML-100K	Ensemble-H	1.0229 ± 0.0006	0.5219 ± 0.0025	0.0783 ± 0.0084	0.2445	0.1236
Amazon	NCF	1.1184 ± 0.0005	0.7662 ± 0.0149	0.3278 ± 0.0116	0.1120	0.0645
Amazon	BayesNCF-v2	1.1213 ± 0.0009	0.6206 ± 0.0046	0.0893 ± 0.0258	0.1289	0.0747
Amazon	MC-Drop-H	1.1196 ± 0.0004	0.6179 ± 0.0066	0.0805 ± 0.0229	0.1253	0.0726
Amazon	Ensemble-H	1.1177 ± 0.0001	0.6115 ± 0.0013	0.0813 ± 0.0085	0.1305	0.0750

**Table 7:** Joint user × item cold-start stratification (temporal split, five seeds).

Dataset	Cell	*n*	BayesNCF-v2 ECE	NCF ECE
MovieLens-1M	cold user × cold item	114	0.303	0.463
MovieLens-1M	cold user × warm item	95,351	0.030	0.140
MovieLens-1M	warm user × warm item	91,928	0.039	0.049
MovieLens-100K	cold user × cold item	350	0.252	0.276
MovieLens-100K	cold user × warm item	15,705	0.081	0.121
Amazon	cold user × cold item	22,488	0.075	0.334
Amazon	cold user × warm item	926	0.247	0.309

**Table 8:** Predictive-interval coverage and PIT diagnostics (temporal split, five seeds, BayesNCF-v2; mean ± std over seeds).

Dataset	cov50	cov80	cov90	cov95	PIT-KS
MovieLens-1M	0.471 ± 0.002	0.794 ± 0.003	0.899 ± 0.003	0.945 ± 0.002	0.043 ± 0.010
MovieLens-100K	0.452 ± 0.009	0.754 ± 0.012	0.884 ± 0.007	0.935 ± 0.004	0.055 ± 0.010
Amazon Digital Music	0.838 ± 0.004	0.871 ± 0.003	0.902 ± 0.001	0.908 ± 0.001	0.350 ± 0.019

**Table 9:** Sensitivity of BayesNCF-v2 to the KL weight λ and the prior scale $\sigma_{p}$ (MovieLens-100K, random split, three seeds).

λ	$\sigma_{p}$	RMSE	ECE	Epistemic Share
$10^{-4}$	0.1	0.9236 ± 0.0009	0.0510 ± 0.0041	1.0%
$10^{-4}$	0.3	0.9244 ± 0.0009	0.0571 ± 0.0041	0.8%
$10^{-4}$	1.0	0.9242 ± 0.0017	0.0602 ± 0.0049	0.7%
$10^{-3}$	0.1	0.9255 ± 0.0006	0.0480 ± 0.0050	2.2%
$10^{-3}$	0.3	0.9241 ± 0.0015	0.0522 ± 0.0027	1.9%
$10^{-3}$	1.0	0.9238 ± 0.0009	0.0571 ± 0.0020	1.7%
$10^{-2}$	0.1	0.9285 ± 0.0005	0.0589 ± 0.0025	5.8%
$10^{-2}$	0.3	0.9268 ± 0.0021	0.0596 ± 0.0256	6.0%
$10^{-2}$	1.0	0.9253 ± 0.0004	0.0353 ± 0.0024	5.2%

**Table 10:** Wall-clock inference time per batch of 512 ratings (CPU) and parameter count.

Method	Parameters	Point (ms/batch)	MC-30 (ms/batch)
NCF	11.0 M	0.51	—
BayesNCF-v2	11.0 M	0.68	19.2
MC-Dropout-H	11.0 M	0.97	20.4
Ensemble-H (5)	55.1 M	3.60	—

## Data Availability

The datasets used and analyzed during the current study are available from the corresponding author upon reasonable request.
